# TM-Free and TM-Catalyzed Mechanosynthesis of Functional Polymers

**DOI:** 10.3390/polym15081853

**Published:** 2023-04-12

**Authors:** Wahab K. A. Al-Ithawi, Albert F. Khasanov, Igor S. Kovalev, Igor L. Nikonov, Vadim A. Platonov, Dmitry S. Kopchuk, Sougata Santra, Grigory V. Zyryanov, Brindaban C. Ranu

**Affiliations:** 1 Chemical Engineering Institute, Ural Federal University, 19 Mira St., 620002 Yekaterinburg, Russia; 2 Energy and Renewable Energies Technology Center, University of Technology—Iraq, Baghdad 10066, Iraq; 3 I. Ya. Postovsky Institute of Organic Synthesis of RAS (Ural Division), 22/20 S. Kovalevskoy/Akademicheskaya St., 620219 Yekaterinburg, Russia; 4 School of Chemical Sciences, Indian Association for the Cultivation of Science, Jadavpur, Kolkata 700032, India

**Keywords:** functional polymers, ball-milling, green chemistry, solid-state chemistry, solvent-free synthesis, TM-catalyzed synthesis, TM-free synthesis

## Abstract

**Highlights:**

- The most representative examples for the TM-free and TM-catalyzed mechano-synthesis of functional polymers are reported;
- The most common applications for the various types of functional polymers are precented;
- The advantage of solvent-free mechanosynthesis over conventional solvent-based synthesis are highlighted;
- In many cases the better performance of the mechanchemically-prepared polymers over those obtained by using conventional methods are demonstrated.

**Abstract:**

Mechanochemically induced methods are commonly used for the depolymerization of polymers, including plastic and agricultural wastes. So far, these methods have rarely been used for polymer synthesis. Compared to conventional polymerization in solutions, mechanochemical polymerization offers numerous advantages such as less or no solvent consumption, the accessibility of novel structures, the inclusion of co-polymers and post-modified polymers, and, most importantly, the avoidance of problems posed by low monomer/oligomer solubility and fast precipitation during polymerization. Consequently, the development of new functional polymers and materials, including those based on mechanochemically synthesized polymers, has drawn much interest, particularly from the perspective of green chemistry. In this review, we tried to highlight the most representative examples of transition-metal (TM)-free and TM-catalyzed mechanosynthesis of some functional polymers, such as semiconductive polymers, porous polymeric materials, sensory materials, materials for photovoltaics, etc.

## 1. Introduction

According to the International Union of Pure and Applied Chemistry (IUPAC), among the 10 chemical innovations that could impact society, reactive extrusion could be the most promising one as it allows chemical reactions to be carried out completely in solvent-free conditions with good E-factors and, thus, with a lower negative impact on the environment [1]. One remarkable example of such a technique is the use of mechanosynthesis, such as grinding, ball-milling etc., as an equal or, in most cases, better alternative to conventional solvent-based conditions for carrying out chemical reactions. In turn, a most remarkable example of such reactions is solvent reduced or solvent-free polymerization, including processes involving coordination bonds [2] under mechanochemical, most commonly ball-milling, conditions. Therefore, the methods for making polymers under grinding/ball-milling conditions, which were formerly used to rupture them, are of wide interest in the chemical community worldwide [3].

It worth mentioning that in past decades, mechanochemical processes/reactions have attracted growing attention due to the green aspects of this type of synthesis [4], especially for the utilization of recyclable materials [5,6], the preparation of biologically active compounds [7,8], the preparation of various types of polymers [9], and the other types of materials [10,11].

In this review, the most representative examples of the mechanosynthesis of functional polymers are presented. The obtained polymers are arranged according to their possible applications and/or polymerization conditions.

## 2. Results and Discussion

### 2.1. Mechanosynthesis of Conductive Polymers 

**Figure 1:**
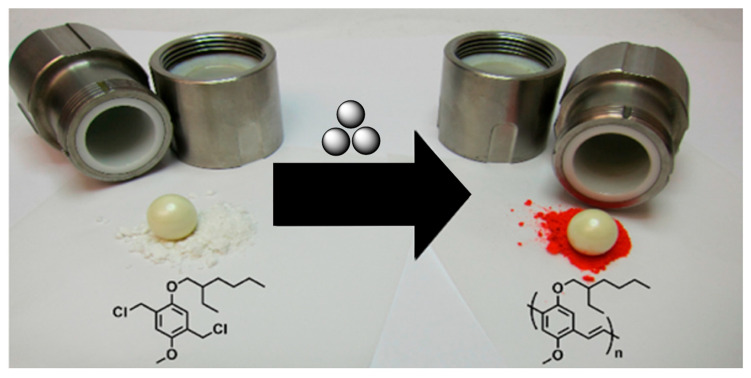
Ball-milling approach to PPVs. Reproduced with the permission of reference [19].

The high electrical conductivity of polyacetylenes and polyethynes were discovered for the first time in the 1970s [12], and since that time these polymers have become promising materials for molecular electronics [13,14,15,16]. On the other hand, poly(*p*-phenylene vinylenes) (PPVs) possess several of such extraordinary attributes, such as tunable optical properties, good reactivity, and high electrical conductivity [17], and, therefore, they are considered as advanced materials for electronics applications, particularly for OLEDs [18]. In 2014, Swager’s group reported the mechanochemical synthesis of poly(phenylene vinylene) [19] (Figure 1). To achieve this, the authors used solid-state base-catalyzed Gilch polymerization in a Retsch vibrational mill and zirconium oxide jar/milling balls. Depending on the amount of milling time, base strength, solid-state dilution, milling frequency, and the size of the milling balls, various polymerization degrees were observed. In the most representative case, PPVs up to a 40 kDa molecular weight were prepared in up to a 70% yield after 30 min milling with 6 eq. of KOtBu. Polymer molecular weights and polydispersity indices were estimated by gel permeation chromatography (GPC) using polystyrenes as standards. 

Among semiconductive polymers, polypyrroles (PPy) are the most attractive ones due to their high conductivity, good stability, and great applicability for various tasks, such as energy storage and transfer, sensory applications [20,21,22,23], and functional membranes [24,25,26]. Electrochemical or chemical oxidative polymerization in water or organic solutions is a common way to prepare polypyrroles. Depending on the type of oxidant and solvent, polypyrroles of various conductivities can be obtained [27,28]. For instance, in an aqueous medium in the presence of ammonium persulfate, PPy with a conductivity not higher than 0.5 S·cm^−1^ was obtained [29]. 

Posudievsky and Kozarenko [30] reported the synthesis of PPy by using solvent-free ball-milling in the presence of ammonium persulfate as an oxidant at an uncommon (for solvent-based approaches) value of the monomer/oxidant (ammonium persulfate) mole ratio equal to two. The PPy was obtained in a high yield, and the greatest level of conductivity (6.5 S·cm^−1^) was achieved.

In a later study [31], the same authors proposed the formation of highly conducting PPy at a high monomer/oxidant (ammonium persulfate) mole ratio via the chain mechanism with the intermediate formation of poly(3-pyrroline) and its further mechanochemical dehydrogenation via both an oxidative (ammonium persulfate) method and under the action of mechanical forces. For the obtained PPy, a conductivity above 5 S·cm^−1^ was achieved only at a relatively low oxidant content in the initial reaction mixture. Based on the TEM data, the PPy with the highest conductivity consisted of up to ∼100 nm nanoparticles with a core–shell structure, with the material of the shell being amorphous and the core being formed by more closely packed polymer macromolecules.

### 2.2. Mechanosynthesis of Polystyrenes and Poly(2-vinylnaphthalene)

Polystyrene (PS) and poly(2-vinylnaphthalene) (PVN) are known to exhibit excimer-induced energy migration properties [32,33], strong excimer fluorescence, and phosphorescence [34,35]. In addition, they are important components for plastic scintillators [36,37,38,39]. 

**Figure 2:**
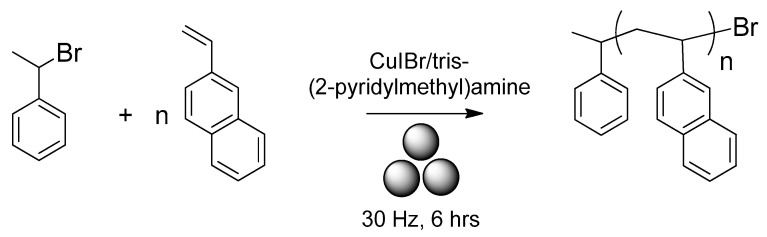
Mechanosynthesis of PVN.

Cho and Bielawski published a paper on the mechanosyntheis of PVN (Figure 2) by using a variant of atom transfer radical polymerization under ball-milling conditions using 2-vinylnaphthalene, phenylethyl bromide (initiator), and CuIBr/tris(2-pyridylmethyl)amine (catalyst) under nitrogen using 10 mm diameter zirconium dioxide jar/balls in a vibrational ball mill at 30 Hz for 6 h [40]. By using semi-logarithmic plot of the monomer concentration vs. time, a linear dependance was observed, with the conversion of the polymerization reaction reaching as high as 97 % after 6 h. In addition, a linear correlation between the polymer MW and monomer conversion was observed, although the experimental Mn, which was estimated by means of size-exclusion chromatography (SEC) using anisole as a standard, was in fact lower than the theoretical one, which was possibly due to premature mechanical degradation. 

It worth mentioning that at a frequency of 10 Hz for 6 h and at the same reagents ratio, no significant polymerization was observed, while at a frequency of 20 Hz, the formation of the desired polymer took place with an Mn of 16.0 kDa, although with a monomer conversion of 50 % and a relatively broad polydispersity (Ð of 3.23). The monomer conversion was analyzed by ^1^H NMR spectroscopy.

In 2007, Hasegawa and co-authors reported the mechanochemically initiated polymerization of styrene via its grinding with talc in SiN_3_ jar/milling balls (8.5 mm) at 24 Hz in a vibrating ball mill [41]. The obtained PSs were isolated as composites of talc particles, that is, the polymer was attached to the talc particles. Their time–conversion studies demonstrated that the polymerization of styrene took place within 1 h, and the conversion of styrene depended strongly on either the grinding time or talc concentration. For instance, a 41% conversion was observed at a talc concentration of 15 wt% with a grinding time of 6 h, while only a 50% conversion was observed after 24 h. The molecular weight of the polymers was measured by means of GPC, and the highest Mn observed was 1.6 × 10^6^ Da. Thus, the authors suggested an efficient way toward forming clay nanocomposites composed of widely used polystyrene possessing attractive thermomechanical properties.

**Figure 3:**
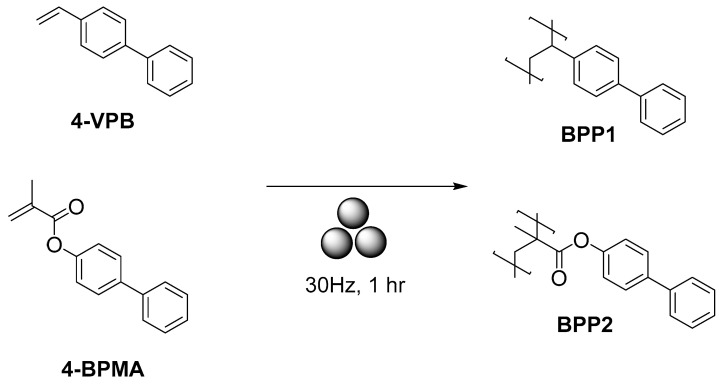
The mechanosynthesis of biphenyl-appended polymers.

Very recently, Kim and co-authors reported a mechanochemical solid-state vinyl polymerization method [42] (Figure 3). In their study, either 4-vinyl biphenyl or 4-biphenyl methacrylate in zircona jars/milling balls (8 mm) were subject to ball-milling in a Retsch Mixing Mill MM400 at 30 Hz for 1 h to produce **BPP1-2** polymers with a 99% conversion. It worth mentioning that at lower speed lower or no conversion was observed. 

The same polymerization was also carried out by using an anionic initiator, such as *sec*-BuLi. According to the authors, the alkyl-anion-promoted polymerization proceeded with excluding radical initiation, and the generally expected features of anionic polymerization, such as molecular weight control and narrow dispersity, were not observed (PDI = 1.25–4.46, Mw = 23.5–309 kDa). The molecular weights of the polymers were determined by SEC analysis using polystyrenes as standards, and the conversion degree was determined by ^1^H NMR spectroscopy. It was suggested that upon ball-milling, the mechanical force fractured the newly formed polymer chains via anionic initiation to generate macroradicals, and these newly formed radicals participated in the polymerization process. In other words, the anionic process was responsible for only the initiation step, and after that, the ball-milling made the radical process become dominant during the polymerization.

### 2.3. Mechanosynthesis of Polyazomethines

The main difference in polyazomethines (PAMs) from polyacetylenes is the presence of C=N moieties, which are isoelectronic to CH=CH ones, and they both have a similar planar molecular structure and maximum absorption peak. Owing to the much easier formation of C=N bonds, PAMs can be suitable alternatives to polyacetylenes. PAMs are widely used in optoelectronic devices [43] such as photovoltaic cells [44,45], electroluminescent devices [46], and electrochromic devices [47,48,49]. Importantly, the dynamic nature of azomethine bonds provides new avenues for using polyazomethines as components for biocompatible and totally disintegrable electronics [50]. However, as with most high-molecular-weight conjugated polymers, the low solubility of polyazomethines in common organic solvents limits their preparation, processability, characterization, and application. Therefore, the mechanosynthesis of PAM is a good alternative to conventional solution-based procedures. 

**Figure 4:**
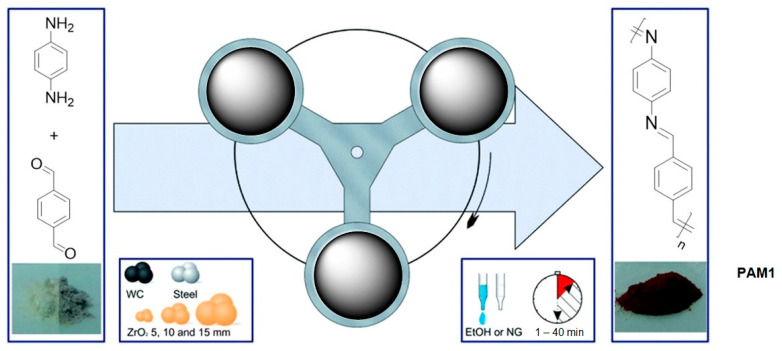
The mechanosynthesis of **PAM1**. Reproduced with the permission of reference [51].

In 2016, Grätz and Borchardt reported [51] the very-first mechanosynthetic approach to polyazomethines, **PAM1**, by mixing equal molar amounts of *p*-phenylenediamine and terephthalic aldehyde in a zirconium oxide milling cup with 22 zirconium oxide milling balls (d10 mm) at 800 rpm for 45 min to produce the desired polymer with Mn = 3010 Da and PDI = 1.36 (based on the data of MALDI-TOF) (Figure 4):

According to the authors, the obtained polymers had a high thermal stability and low optical bandgaps (λ_max_ = 456 nm). The influence of the milling ball size and material was investigated, and the tungsten carbide ones gave the highest conversion, but all the materials gave higher yields compared to solution polymerization due to the absence of the influence of the solvent. The formation of **PAM1** could be easily monitored by solid-state IR based on the appearance of a vibration of the C=N group at 1609 cm^−1^ as well as the disappearance of the vibrations of the carbonyl and amine groups at 1686 cm^−1^ and 1514 cm^−1^, respectively. All the above-mentioned information strongly supports the effectiveness of mechanosynthesis for the preparation of polyazamethines. 

**Figure 5:**
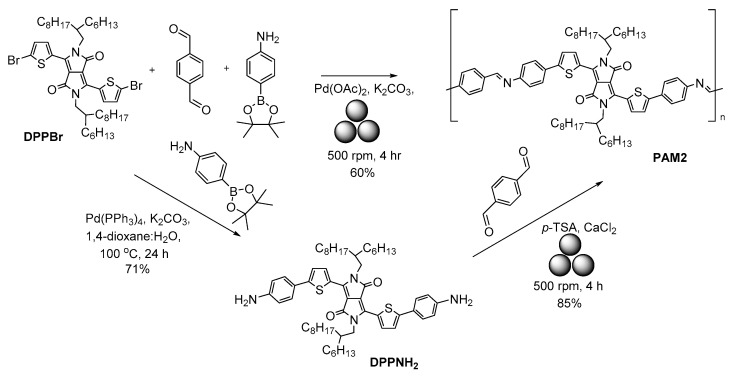
The mechanosynthesis of DPP-based polyazamethine, **PAM2**.

We recently reported on the mechanosynthesis of diketo-pyrrolopyrroles (DPPs)-based azomethine polymers [52] (Figure 5). Two synthetic strategies were used. In the first approach, a combination of Pd(OAc)_2_-catalyzed Suzuki cross-coupling and a condensation reaction was used, and dibromo-substituted **DPPBr** reacted with 4-(4,4,5,5-tetramethyl-1,3,2-dioxaborolan-2-yl)aniline and terephthalic aldehyde in the presence of potassium carbonated in a stainless-steel jar/milling balls upon ball-milling in a Retch Planetary Mill PM100 at 500 rpm for 4 h to produce the polymer **PAM2** in a 60 % yield. In the second approach, owing to the wide use of DPP-based materials for biological applications [53], a cytotoxic Pd-free synthesis was developed by reacting aniline-appended **DPPNH_2_** with terephthalic aldehyde in the presence of *p*-toluenesulphonic acid (*p*-TSA) and with an excess of CaCl_2_ as a dehydrating agent to produce target polymer **PAM2** in a yield as high as 85%.

**Figure 6:**
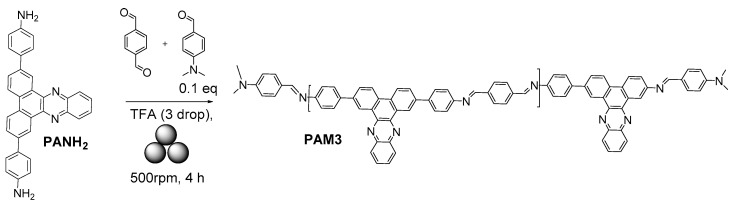
The mechanosynthesis of dibenzo[*a,c*]phenazine-containing polymer, **PAM3**.

By using similar conditions, an azamethine-linked dibenzo[*a,c*]phenazine-containing polymer **PAM3** was prepared [54] (Figure 6). The Mn = 5365 Da was calculated by using ^1^H NMR end-group analysis. 

### 2.4. Mechanosynthesis of Biopolymers and (Bio)Degradable Polymers

In past decades, closed-loop plastic recycling, as a process by which a product or material can be used and then turned into a new product or converted back to raw material without losing its properties during the recycling process, has gained wide interest worldwide [55,56,57,58,59,60,61,62,63]. For instance, dynamic covalent polymers, such as vitrimers, have been proposed as a possible alternative to non-recyclable polymers [64,65,66,67,68,69,70]. It is worth mentioning that the depolymerization of vintimers is still a challenge as it commonly requires high temperatures and, in many cases, it does not produce the starting monomers but produces short oligomers. 

**Figure 7:**
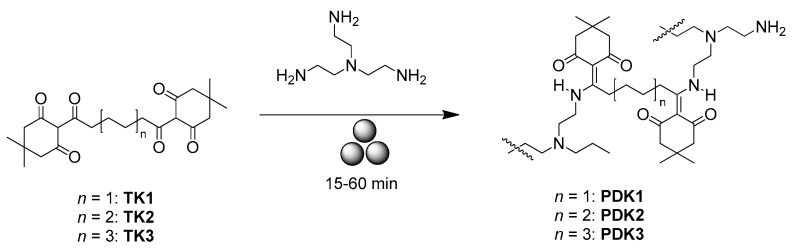
The mechanosynthesis of poly(diketoenamine)s **PDK1-3**.

Christensen et al. suggested using diketoenamine dynamic bonds and mechanical force for the closed-loop recycling of plastic poly(diketoenamine)s (PDKs) [71]. In their work, **PDK1-3** was prepared in high yields (95%) via simple polycondensation reactions between ß-triketones and either aromatic or aliphatic amines by using a stainless-steel jar/milling balls in a SPEX SamplePrep 8000 Mixer/Mill for between 15 and 60 min (Figure 7).

In addition, the authors reported a recycling process (depolymerization) for the polymer to recover the used monomers. A disintegration process occurred during room-temperature hydrolysis in aqueous strong acid solutions to collect pure triketones, while the amine monomers were recovered by a regenerative resin-based process. Interaction with sulfuric or hydrochloric acids (5.0 M) during 12 h recovered the pure monomers in a more than 90% isolated yield. Moreover, it was confirmed experimentally that the presence of various types of polymers and plastics, such as poly(ethylene terephthalate) (PET), nylon-6,6 (PA), polyethylene (PE), poly(vinyl chloride) (PVC), and polycarbonate (PC), as well as dyes, inorganic substances, etc., did not interfere with the regeneration process, thus confirming the high ability of PDKs to be recovered in a high selectivity. This degradation/regeneration process represents an efficient closed-loop recycling method of a polymer with potential applications in biodegradable materials.

**Figure 8:**
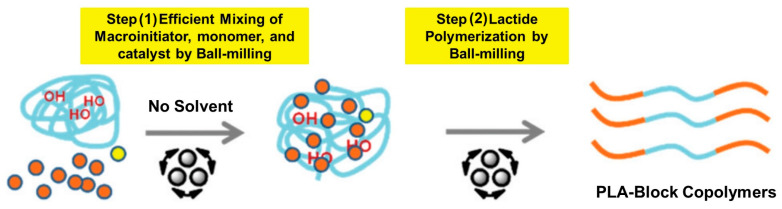
The mechanosynthesis of poly(lactic acid) block co-polymers. Reproduced with the permission of reference [73].

Poly(lactic acid)(PLA)-based polymers are another type of readily degradable materials for bioplastics [72]. In 2019, Lee and co-workers reported the mechanochemical synthesis of PLA block copolymers [73] (Figure 8). 

In the most representative case, the authors were able to prepare PLA-PEO-PLA block copolymer via the reaction of *D*-Lactide, polyethylene oxide 6000, and DBU in a stainless-steel milling container/milling ball (12 mm diameter) in a Retsch Mixer Mill MM 400 at 20 Hz for 1 h to produce the target polymer with Mn = 17.9 kDa (PDI = 1.33, based on GPC) with a >99% conversion. By using a similar approach (7 mm milling ball), other di- and three-block co-polymers, such as PLA4000-PεDL4600-PLA4000 (92%, 19.9 kDa, PDI = 1.52), PLA4000-PδDL4400-PLA4000 (88%, 10.7 KDa, PDI = 1.41), PεCL4800-PLA4000 (89%, 11.3 kDa, PDI = 1.20), PLA2000-PTHF2900-PLA2000 (94%, 8.89 kDa, PDI = 1.55), and PLA4000-PTHF2900-PLA4000 (83%, 13.2 kDa, PDI = 1.26), were successfully prepared. Due to the biodegradability of PLA, these polymers could have biomedical applications.

**Figure 9:**
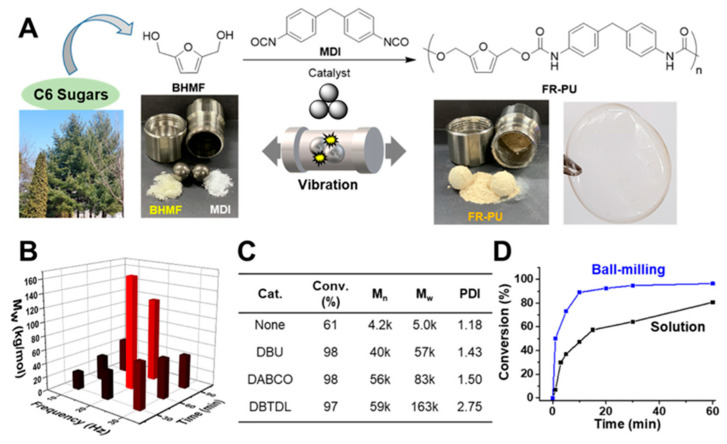
(**A**) Schematic illustration of PU synthesis using diols derived from biomass. Screening of ball-milling polymerization with BHMF and MDI (**B**) by controlling the frequency and the reaction time and (**C**) with several catalysts. The reaction with the DBTDL catalyst at 20 Hz for 60 min yielded the highest Mw PU. (**D**) Comparison of conversion achieved with ball-milling vs. solution synthesis of PU as a function of the reaction time. Reproduced with the permission of reference [77].

Along with 2,5-furandicarboxylic acid (FDCA) [74,75] and 5-hydroxymethylfurfural (HMF) [76], 2,5-bis(hydroxymethyl)furan (BHMF) can be considered as one of the important bio-based building blocks for green chemistry and as an important monomer for biopolymers. In 2020, Oh and co-authors [77] reported a facile mechanochemical synthesis of BHMF-derived eco-friendly polyurethanes (PUs) (Figure 9). To achieve this, BHMF was reacted with di-isocyanates in the presence of either DBTDL, DABCO, or DBU upon ball-milling in a vibration mill. As a result of these two-component mechanochemical polymerization reactions, a variety of BHMF-containing PUs were obtained with a Mw that varied from 5 to 163 k with PDI = 1.18–2.75. According to the authors, these PUs were flexible (Tg = 96 °C) and thermally stable (Td = 197 °C). In addition, three-component mechanochemical polymerization was carried out (with BHMF and equimolar amounts of either aliphatic diols or diamines) to produe PU co-polymers with a wide variation in the polymer properties, such as the glass transition temperature and molecular weight (Mw = 13–111 kDa, PDI = 1.25–1.75).

### 2.5. Mechanosynthesis of Polyphenylenes

Functional polyphenylenes (FPPs) are one of the hottest topics for use in organic electronics [78,79] and photovoltaics [80]. The most common methods for the preparation of FPPS involve the Friedel–Crafts [81], Ullmann, and Suzuki cross-coupling reactions. However, the low solubility of FPPs is themain drawback for their preparation by solvent-based methods. Therefore, some synthetic approaches, such as polymerization on a surface [82] and Friedel–Crafts post-modification [83], are used. 

**Figure 10:**
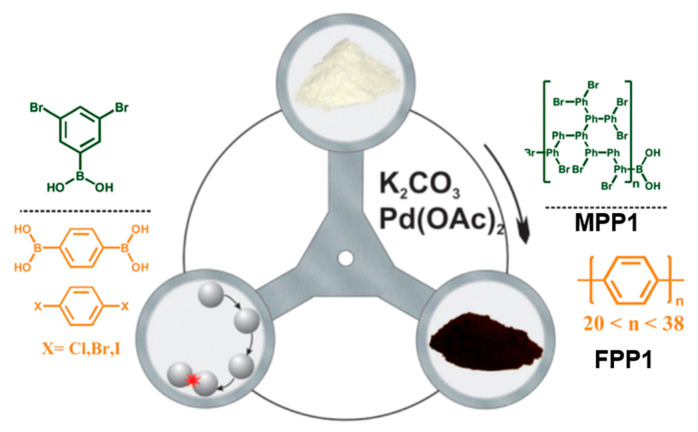
Mechanosynthesis of polyphenylenes and hyperbranched derivatives. Reproduced with the permission of reference [85].

Borchardt’s group reported a series of works on mechanochemical Suzuki cross-coupling polymerization [84,85] (Figure 10). In the most representative case, 1,4-dibromobenzene reacted with 1,4-phenyldiboronic acid in the presence of palladium acetate and potassium carbonate in a zirconium oxide jar with 22 zirconium oxide grinding balls (10 mm) in a Fritsch Pulverisett 7 planetary ball mill at 800 rpm for 30 min to afford the linear polyphenylene (**FPP1)** with an outstanding degree of polymerization (DP) of 164. Among all the aryl halides used, bromide was found to be the best functional group, leading to the highest DP and yield while also showing a defined structure of the polymers. According to the authors, the atom economy of the Suzuki reaction (38%) was identical for conventional solvent-based and mechanochemical approaches. However, the conventional solvent-based process proceeded with low mass productivity of 1.4% (due to the solvents present). Finally, the mechanochemical approach provided three-times-as-high yields (10.6%). 

In addition to linear polymers, by using 3,5-dibromophenylboronic acid, the same approach was used to prepare a microporous hyperbranched polymer (**MHP1**) with a high temperature resistance and high yields in short reaction times.

Among the FPPs, polyfluorenes (PFs) [86] and their copolymers [87,88,89] exhibited advanced optoelectronic properties due to the influence of micro- and macrostructural organization in a solid-state and/or polymer film [90,91,92,93,94,95,96,97]. Therefore, the method of preparation of PFs can strongly influence their properties and performance. 

**Figure 11:**
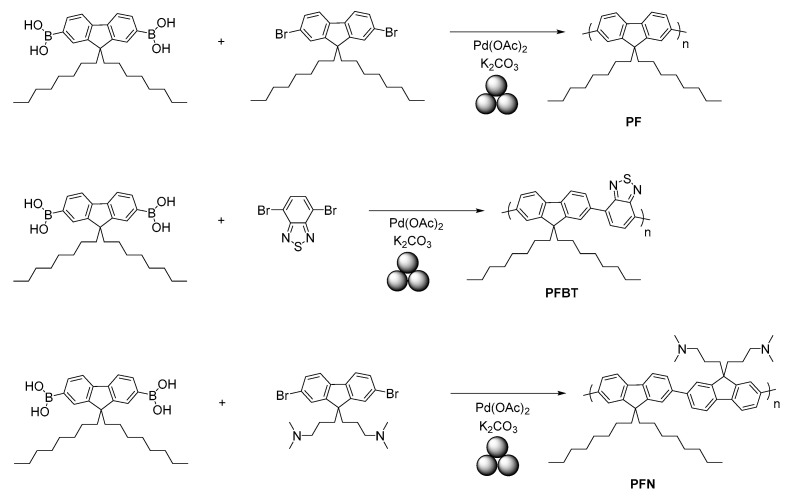
Mechanosynthesis of polyfluorenes.

Very recently, Nelson’s group developed [98] a mechanochemical Suzuki polymerization method to prepare polyfluorene-conjugated polymers, such as poly(9,9-di-*n*-octylfluorenyl-2,7-diyl) (**PF**), poly(9,9-dioctylfluorene-alt-benzothiadiazole) (**PFBT**), and poly[(9,9-bis(3′-(*N*,*N*-dimethylamino)propyl)-2,7-fluorene)- alt-2,7-(9,9-dioctylfluorene)] (**PFN**) (Figure 11). The authors provided extended research on optimizing the reaction conditions such as the milling frequency and time and catalyst loading on the polymer molecular weights, dispersity, and yield. It was found that the Pd catalyst loading played a key role, while the milling time and frequency played a less important role. 

Moreover, a polyelectrolyte, PFN-Br, was developed using solvent-assisted ball-milling polymerization from a PFN polymer during mechanochemical quaternization at the terminal amino groups. This material could have a potential application as an electron-interface-layer material in OFETs, OLEDs, OPVs, and perovskite solar cells to improve the interfacial properties.

### 2.6. Mechanosynthesis of Polyanylines and Polyamines

Polyanilines (PANIs) are the most famous conductive polymers [99,100,101] due to the simplicity of their preparation via connecting the 1,4-coupling of aniline monomer parts, environmental stability, ability to be doped by protonic acids, and, finally, ability to exist in different oxidation states, such as (a) leucoemeraldine, (b) emeraldine (salt/base), and (c) pernigraniline. PANIs and their derivatives/co-polymers are extensively applied in rechargeable batteries, photovoltaic cells, gas separation membranes, chemical sensors, anti-corrosion coatings, microwave absorption electromagnetic interference shielding, electrodes and supercapasitors, reagents for photothermal therapy, etc. [102,103,104]. 

Zhou and co-authors reported a PANI synthesis method by using the interaction of aniline sulphate with ammonium persulphate in a pan mill (600 rpm) and, for comparison, by means of mortar grinding for 40 min [105]. The authors observed that for two pan mill cycles, the molecular weight of PANI was lower than for the mortar-grinded mixture, whilst it was almost equal after ten cycles, and twice as large after twenty cycles. 

In 2011, a PANI mechanosynthesis method was reported [106] by mixing anilinium hydrochloride with different oxidants (ammonium persulphate, FeCl_3_, and AgNO_3_) with a mortar (5 min) with the following treatment of the obtained powder with air. According to the authors, the PANI formed with ammonium persulphate in 24 h, while after one week with FeCl_3_ and AgNO_3_, only short oligomers and branched non-conductive polymers were obtained using Fe^3+^ or Ag^+^. 

Posudievsky and co-authors reported the synthesis of highly conductive PANI (22.3 S/cm) by means of grinding anilinium chloride and ammonium persulphate in a planetary mill by using an agate jar and milling balls at 300 rpm [107]. For comparison, the PANI was obtained via a solvent-based procedure. Even though the molecular weights of both polymers were comparable, a better conductivity was observed for the PANI obtained by using methanosynthesis. This difference was attributed by the authors to the influence of mechanical stress on the polymer during its mechanochemical preparation, and an increased conductivity of the PANI obtained via the solvent-based procedure by post-synthesis mechanochemical treatment was observed.

It worth mentioning that, earlier, Huang and coauthors [108] reported a PANI synthesis method using the interaction between anilinium chloride and ammonium persulfate in a stainless-steel jar using stainless-steel milling balls (5–10 mm) at 600 rpm in a Pulverizette 7 planetary micromill for 1 h. Highly conductive PANI was obtained in a 65% yield at an ammonium persulfate:ammonium chloride ratio = 1:2. A conductivity of 0.01 S/cm was observed. 

Bhandari and Khastgir reported the mechanosynthesis of ultra-long nanofibrous PANI by means of grinding anilinium chloride and ammonium peroxydisulphate in the presence and absence of citric acid (as a dopant) by using a mortar and pestle for 30 min [109]. According to the authors, citric acid influenced the morphology of the PANI via hydrogen bonding and provided the doping of PANI, while in the absence of citric acid, the PANI was “undoped”. In addition, this in situ doping dramatically influenced the electrochemical behavior of the PANI. 

**Figure 12:**
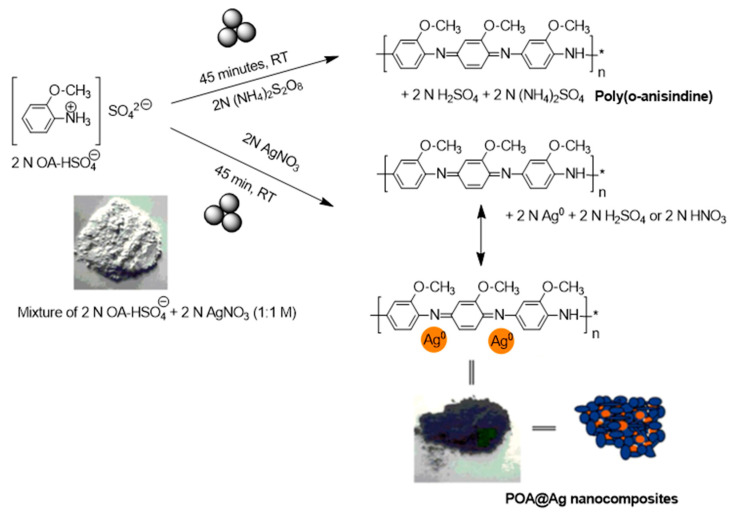
Mechanosynthesis of **POA** and **POA@Ag**, “*” represents a multiplication sign. Reproduced with the permission of reference [110].

In 2021, a mechanochemical oxidative polymerization method using an OMe derivative of PANI, poly(*o*-anisidine) (POA), and POA-protected silver nanoparticles, POA@Ag, was reported [110] (Figure 12). As a first step, the authors subjected anisidinium sulphate (OA-HSO_4_) to mechanopolymerization as a monomer to produce POA in the presence of ammonium persulphate as an oxidant. In the second step, POA was formed in situ in the presence of AgNO_3_ as both an oxidant and a metal precursor to produce POA-protected silver nanoparticles, **POA@Ag**. In this case, an equimolar amount of OA-HSO_4_ and AgNO_3_ (2 mM, 0.34 g) were hand-ground in mortar with a pestle for 10 min, resulting in the formation of a slurry, which in 15–45 min converted into a pale green color, with the final product being the green-colored emeraldine salt. Ag nanoparticles were also immobilized in the obtained polymer matrix. Based on electrochemical studies, the interconversion of POA between the leucoemeraldine ↔ emeraldine and emeraldine ↔ pernigraniline redox transformation and the redox responses of the AgNPs was observed. The authors suggested the potential application of **POA@Ag** as an electrocatalyst. In addition, the electrochemical response of **POA@Ag** toward dopamine via cyclic voltammetry (CV), differential pulse voltammetry (DPV), and chronoamperometry was observed with the electrochemical stability of **POA@Ag** for the dopamine determination being in the 10–130 μM range and with a limit of detection (LOD) as low as 2.8 μM. In a chronoamperometry-based method, dopamine was detected in the range of 5–45 μM (0.83 μM LOD). Finally, a **POA@Ag**/GCE-modified electrode for the determination of dopamine from mixtures was prepared, which was able to operate at a 160 mV potential difference with repeatability after 15 and 30 days of immersion.

It worth mentioning that mechanosynthetic approaches are widely used for the preparation of PANI-based nanocomposites via the in situ formation of PANI upon ball-milling or grinding anilines and oxidants (if needed) with different additives, such as porous clays, carbon nanotubes, metal, and oxide nanoparticles, etc. [111]. 

**Figure 13:**
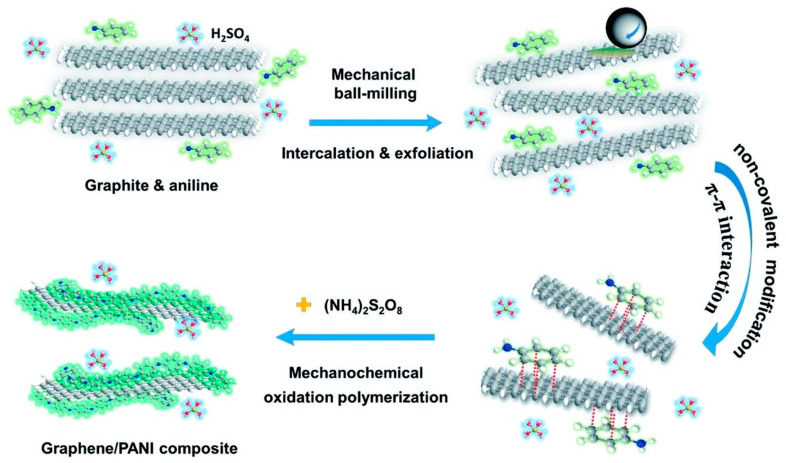
Mechanosynthesis of graphene/PANI composites. Reproduced with the permission of reference [112].

Very recently, Yang, He, and co-authors reported [112] (Figure 13) an efficient method for the construction of graphene/PANI composites via a one-pot high-energy ball-milling process. In this process, aniline molecules acted as both the intercalator for the exfoliation of graphite and the monomer for mechanochemical polymerization into PANI clusters on the in situ exfoliated graphene sheets. The obtained graphene/PANI composite electrode delivered a large specific capacitance of 886 F·g^−1^ at 5 mV·s^−1^ with a high retention of 73.4% at 100 mV·s^−1^. In addition, a high energy density of 40.9 W·h·kg^−1^ was achieved by the graphene/polyaniline-based symmetric supercapacitor at a power density of 0.25 kW·kg^−1^, and the supercapacitor also maintained 89.1% of the initial capacitance over 10,000 cycles. 

**Figure 14:**
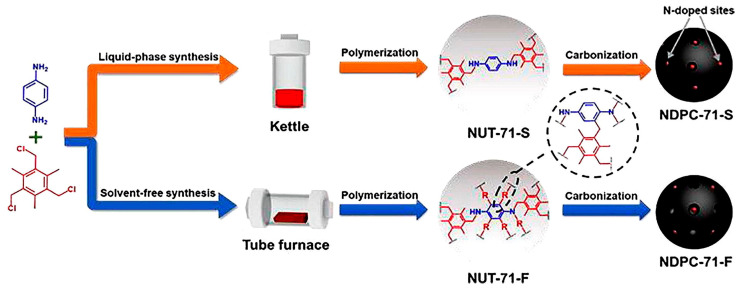
Mechanosynthesis of *N*-connected polymers. Reproduced with the permission of reference [113].

Along with PANIs, anilines can be involved in the preparation of other polyamines by using mechanosynthesis. For example, Lou and co-authors reported [113] (Figure 14) a mechanosynthesis method of highly crosslinked *N*-connected polymers by using solvent-free and mechanochemical conditions (**NUT-71-F**) and, for comparison, a conventional solvent-based approach (**NUT-71-S**). According to the authors, upon mechanical grinding in a mortar, **NUT-71-F** exhibited a higher reaction yield in comparison with **NUT-71-S** (70.8% vs. 49.8%) due to the greater crosslinking degree and different linkage ways. 

In addition, by the carbonization of both polymers at different temperatures (500 °C, 600 °C, 700 °C, and 800 °C, respectively), the authors constructed *N*-doped porous carbons (NDPCs) and evaluated their affinity and selectivity for a gaseous N_2_/CO_2_ (85/15, *v*/*v*) mixture. It was found that **NDPC-71-F** (from mechanochemically prepared **NUT-71-F**) possessed a higher specific surface area and a larger pore volume compared to **NDPC-71-S** (obtained from **NUT-71-S**) and could separate CO_2_ from a N_2_/CO_2_ mixture more efficiently. For instance, the pore size varied from 0.52 to 0.70 nm in **NDPC-71-F-700**, while it only varied from 0.43 to 0.51 nm in **NDPC-71-S-700**. It should be noted that increasing the carbonization temperature to 800 °C excessively enlarged the pore size, making them unable to effectively capture CO_2_.

Moreover, no significant reduction in the CO_2_ adsorption capacity of **NDPC-71-F-700** was detected after six regeneration experiments, which is crucial for potential practical application.

### 2.7. Mechanosynthesis of Organic Porous Polymers

Organic porous polymers (OPPs) have several unique features, such as high surface/contact surface area, highly rigid permanent porous structure, low skeletal density along with good chemical and thermodynamic stability, and, thus, porous polymers have a wide range of applications [114,115], including catalytic applications [116,117,118,119,120], gas storage [121,122,123,124,125], and gas separation [126,127]. The most convenient approach to creating OPPs is the so-called bottom-up building concept, which involves the stepwise building of the desired material by using monomer units containing various functionalities by using either TM-catalyzed transformations, such as cross-coupling reactions [128], Friedel–Crafts alkylations [129], and cyclotrimerization reactions [130], or TM-free approaches, such as Schiff base formation reactions [131], amidisation reactions [132], etc. In these approaches, in order to achieve permanent micro- and mesoporosity, an initial intensive mixing is crucial. In addition, shrinkage of the obtained material upon the drying step takes place after the removal of the absorbed organic vapors or liquids. Finally, the low solubility of most OPPs remains the main challenge, and in some cases the solution-based procedures may suffer from the precipitation of reagents/products to produce OPPs with a low degree of polymerization [133]. 

The ball-milling polymerization process allows the obtaining of polymeric porous materials without solvents, has wide applicability, has an easy synthetic set-up (ball-milling jar), is low-cost, has a simple pre- and post-treatment, and has little or no influence and dependance on the environment. Therefore, ball-milling-assisted polymerization may be considered to be a versatile tool for the synthesis of OPPs. 

**Figure 15:**
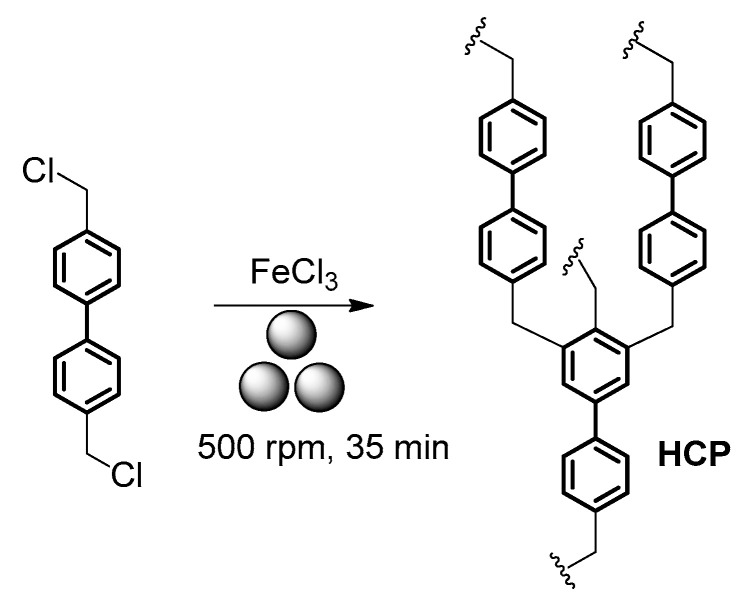
Mechanosynthesis of **HCP**.

For instance, Grätz, Borchardt, and coauthors [134] reported a mechanochemical synthesis method of producing hyper-crosslinked polymers (**HCP**), which may be considered as some of the most promising OPP candidates [135], by using a solvent-free Friedel–Crafts alkylation reaction involving 4,4′-bis(chloromethyl)-1,1′-biphenyl (Figure 15). In a typical procedure, 4,4′-bis(chloromethyl)-1,1′-biphenyl) and FeCl_3_ were milled in a zirconium oxide milling vessel filled with 22 zirconium oxide balls (10 mm) in a Fritsch Pulverisette 7 mill at 500 rpm for 35 min to produce a porous polymer with BET surface areas of up to 1720 m^2^·g^−1^ and pore volumes of up to 1.55 cm^3^·g^−1^ with a narrower pore size distribution compared to their solvent-based analogues. The obtained polymer exhibited a preferable adsorption of benzene vapors over cyclohexane, which was, according to the authors, due to the strong π–π interactions with the aromatic framework.

**Figure 16:**
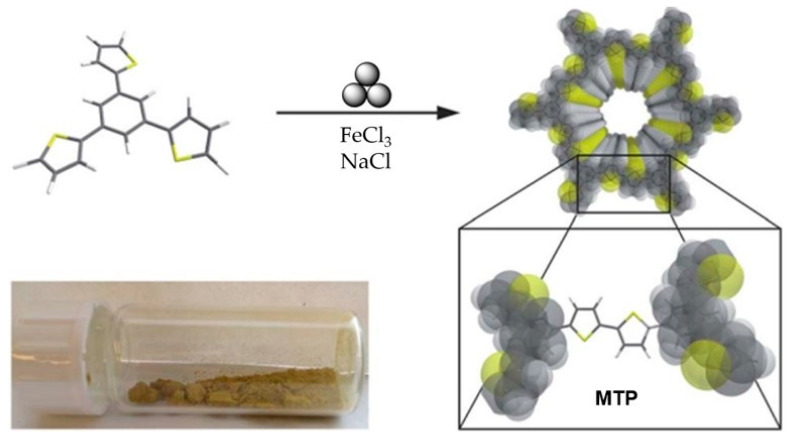
Mechanosynthesis of **MTP**. Reproduced with the permission of reference [136].

Later, the same group reported [136] a more advanced mechanosynthesis method of producing a microporous thiophene polymer (**MTP**) via oxidative polymerization in the presence of NaCl as a bulking material (Figure 16). In a typical procedure, 1,3,5-tris(2-thienyl)benzene, FeCl_3,_ and the inert bulking material NaCl (to control the abrasion [137]) were mixed in a stainless-steel grinding jar with 22 grinding balls (10 mm) at 400 rpm in a Fritsch Pulverisette 7 premium line planetary ball mill for 60 min. In the optimization studies, the ball size (10–15 mm) and milling speed (400–600 rpm) were optimized for the highest yields (up to 98%). The obtained **MTP** exhibited a specific surface area of 1850 m^2^·g^−1^ and a pore volume of 0.95 cm^3^·g^−1^ with a narrow pore size distribution and with one major pore at 1.6 nm. According to the authors, the observed surface area was almost twice as high as the reported values for the solution-based process. The obtained material absorbed Ar and N_2_, and the authors did not observe the typical swelling behavior, which was most probably due to the higher degree of polymerization and crosslinking and therefore the more rigid structure of the polymer.

In addition, to prove the generality of the developed strategy of mechanosynthesis of polymers, the authors applied our procedure to the synthesis of previously synthesized polycarbazoles [138] via FeCl_3_-catalyzed polymerization in a vibrational ball mill (Retsch mixer-mill 400 at 30 Hz for 0.5 h) to produce a microporous carbazol-based polymer starting from 1,3,5-tri(9-carbazolyl)-benzene as a promising candidate for CO_2_ storage. The polycarbazole obtained by using the above-mentioned advanced protocol exhibited a surface area of 1710 m^2^·g^−1^, which exceeded the previously reported [139] values by a factor of two.

**Figure 17:**
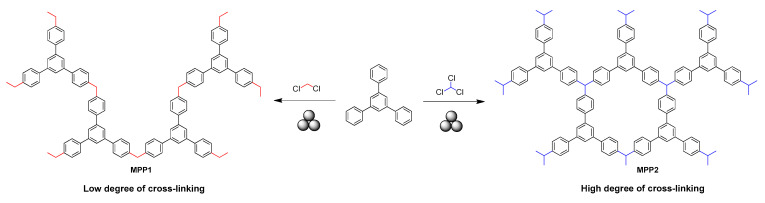
Mechanosynthesis of DCM- and CHCl_3_-linked OPPs (**MPP1-2**).

Very recently, the same group reported the mechanosynthesis of another microporous polymer (**MPP1-2**) [140] by using a Friedel–Crafts alkylation of 1,3,5-triphenylbenzene with two organochloride cross-linking agents, dichloromethane (DCM) (**MPP1**) and chloroform (CHCl_3_) (**MPP2**), respectively (Figure 17). In a typical protocol, TPB and DCM or CHCl_3_ in the presence of AlCl_3_ were milled in a zirconium oxide milling jar with 22 milling balls (10 mm) for 1 h at 30 Hz to produce the target polymers. DCM-linked polymers were found to be flexible and extremely sensitive towards parameter changes, which even enabled the synthesis of a polymer with a BET surface area of 1670 m^2^·g^−1^, while the CHCl_3_-linked polymers were more rigid with a high porosity (the surface area was found to be 1280 m^2^·g^−1^). Based on green metrics calculations, the mechanosynthesis had an advantage over the solvent-based one in terms of the reaction time (0.5 h vs. 48 h), mass intensity (4 vs. 31–37), mass productivity (23 vs. 3), and overall E-factor (1.8–2 vs. 30–36). 

**Figure 18:**
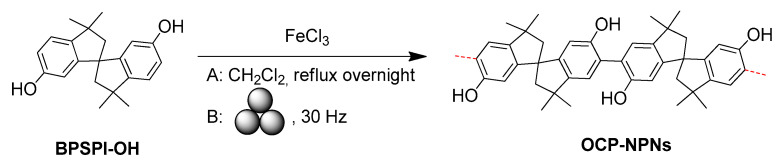
Synthesis of OCP-NPN1-3.

Ladder-like polymers of intrinsic microporosity (PIMs) with contorted sites have been reported as a family of soluble porous polymers and have been successfully utilized in membrane-based gas separations [141,142]. In these polymers, ladder-like monomer units are the main contributor for achieving the high porosity of the resulting polymers. Thus, Tian, Liu, Jin, Dai, and coauthors [143] reported a solvent-free mechanosynthesis method of producing a novel family of soluble fluorescent nanoporous polymer networks based on 3,3,3′,3′-tetramethyl-2,2′,3,3′-tetrahydro-1,1′-pirobi[indene]-6,6′-diol (**BPSPI-OH**). The authors used either a solvent-mediated FeCl_3_-initiated oxidative coupling reaction (A) or mechanochemical approach (B) (Figure 18). As a result, three polymers, **OCP-NPN1-3,** were obtained. 

According to the authors, for the polymer **OCP-NPN-1**, prepared under ball-milling conditions in the presence of FeCl_3_, the Brunauer–Emmett–Teller (BET) surface area was lower than that of the polymer prepared via the solvent-mediated method (470 vs. 834 m^2^·g^−1^). Moreover, dissolving and re-precipitating the polymer from CH_2_Cl_2_ resulted in a decrease in the BET surface area (205 vs. 470 m^2^·g^−1^) due to the possible swelling of **MC-OCP-NPN-1**. However, the BET value could be increased to 319 m^2^·g^−1^ by means of the repeated dissolution and re-precipitating process in CH_2_Cl_2_:EtOH = 1:4. For the ball-milling approach, the FeCl_3_ content was critical, and by increasing the FeCl_3_ amount to 4 mol. eq. the authors obtained **OCP-NPN-3** with a BET surface area of 733 m^2^·g^−1^. In experiments with gas absorption, the **MC-OCP-NPN-1** sample showed selectivity to CO_2_ over CH_4_ (20.9 cm^3^·g^−1^ vs. 5.8 cm^3^·g^−1^ of uptake). Finally, a **MC-OCP-NPN-1** mixed-matrix membrane was prepared, and this matrix membrane exhibited an efficient CO_2_/CH_4_ separation with a high CO_2_ permeability of 675 and a CO_2_/CH_4_ selectivity of 25.

**Figure 19:**
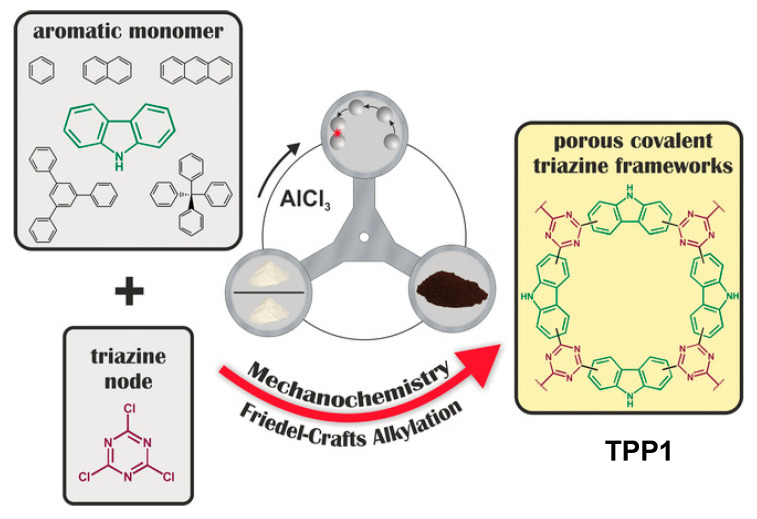
Mechanosynthesis of **TPPs**, “*” means collision event between the milling balls. Reproduced with the permission of reference [157].

Covalent 1,3,5-triazine-based frameworks are another promising scaffold for constructing porous polymers with surface areas >3200 m^2^/g [144] combined with high chemical and thermal stability up to 700 °C [145,146], and they are commonly prepared via nitrile cyclotrimerization approaches [147,148,149]. 1,3,5-Triazine-based porous polymers have a wide range of applications, such as electrode materials in supercapacitors [150] or lithium-sulfur batteries [151,152,153], materials for CO_2_ capture [154,155,156], etc. Another approach to such polymers involves an AlCl_3_-mediated Friedel–Crafts alkylation method by using cyanuric chloride as a core unit [154]. Lübken and Borchardt recently reported a mechanochemical approach to producing *s*-triazine-based porous polymers (**TPPs**) [157] by using an *s*-triazine node (cyanuric chloride) and various aromatic coupling partners upon ball-milling in a planetary ball mill in the presence of stoichiometric amounts of AlCl_3_ as an activating reagent and ZnCl_2_ as a bulking agent (Figure 19).

In a typical procedure, cyanuric chloride, AlCl_3_, and ZnCl_2_ were reacted in either a tungsten carbide grinding jar with 22 balls (10 mm) or in a zircon oxide grinding jar with 22 tungsten carbide balls (10 mm) in a Fritsch Pulverisette 7 planetary ball mill at 800 rpm. In model experiments using carbazole, the authors observed a 32 % yield of polymer **TPP1** after 15 min and a 98% yield after 60 min, and the porosity of the material remained constant from this point on (740 m^2^·g^−1^ due to N_2_ physisorption). According to a BET model, the specific surface area for **TPP1** was 570 m^2^·g^−1^, and a sharp pore size distribution was observed, showing two micropores of 0.5 nm and 1.0 nm, respectively. By using optimized reaction conditions, the authors obtained other polymers by using benzene (**TPP2**, specific surface area of 170 m^2^·g^−1^, 0.20 nm pore size, 5% yield), naphthalene (**TPP3**, specific surface area of 110 m^2^·g^−1^, 0.17 nm pore size, 2% yield), and tetraphenylmethane (**TPP4**, specific surface area of 390 m^2^·g^−1^, 0.43 nm pore size, 3% yield). 

**Figure 20:**
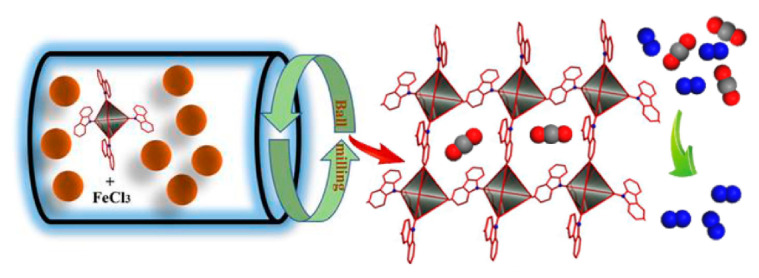
Mechanosynthesis of **CzPP**. Reproduced with the permission of reference [158].

A carbazole-based OPP (**CzPP**) with a high surface area and excellent stability was reported as a promising porous material to capture and separate CO_2_ under mild conditions [158] (Figure 20). To achieve this tetrakis(4-(9H-carbazol-9-yl)phenyl)methane and FeCl_3_ were reacted in an agate tube with ball milling for 2 h to produce **CzPP** in a 91% yield. As calculated by DFT, the median pore width for C was 0.75 nm, and the total pore volume was 0.63 cm^3^/g. As for gas sorption, **CzPP** demonstrated selectivity toward CO_2_ over N_2_ in a binary gas mixture. 

**Figure 21:**
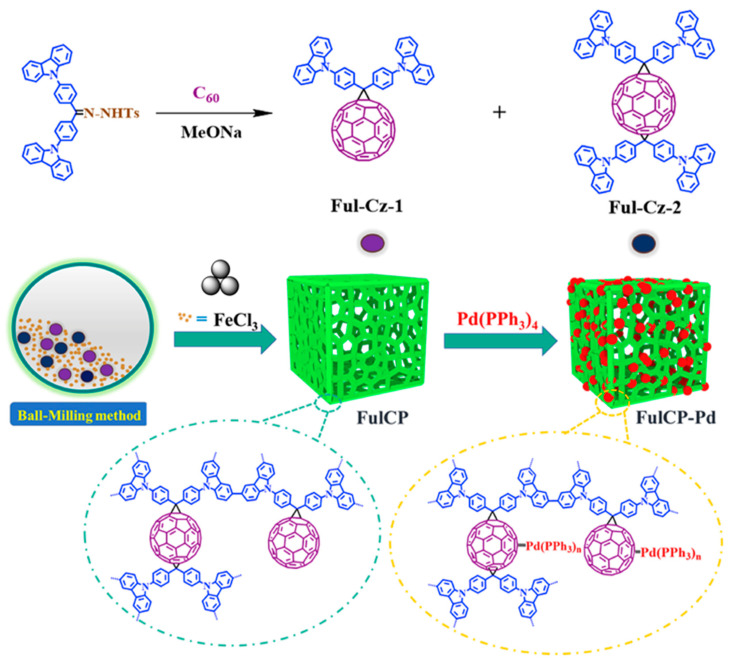
Mechanosynthesis of **FulCP**. Reproduced with the permission of reference [159].

Another type mechanochemically prepared CzPP was reported by Wang and co-authors [159] (Figure 21). In addition to carbazole, a fullerene moiety was introduced into the polymer structure. In a typical case, di-(9H-carbazol-9-ylphenyl) methylene fullerenes, **Ful-Cz-1** and **Ful-Cz-2**, were reacted together with FeCl_3_ in a stainless-steel jar with stainless-steel balls in a Retsch mixer-mill 400 at 30 Hz for 30 min to procude the polymer **FulCP** in an 82% yield. The solution method required 12 h with a tedious purification method. The amount of FeCl3 had a great influence on the specific surface area of the products prepared by the solvent method, but it did not affect the specific surface area of products prepared by the ball-milling method. The pore size distribution of the mechanochemically prepared **FulCP** was mainly 0.64 nm, which implied a microporous nature, and the dominant pore size distribution peaks for the **FulCP** prepared by the solution-based method were at 0.54 and 1.18 nm. The Brunauer–Emmett–Teller specific surface area of the **FulCP** prepared by the ball-milling method (1015 m^2^·g^−1^) was higher than that produced by traditional solvent method (920 m^2^·g^−1^). The obtained polymer was further used to prepare a **FulCP**-supported palladium complex (**FulCP-Pd**) as a heterogeneous catalyst in a deallylation reaction. The conversion efficiency of **FulCP-Pd** with different substrates ranged from 76% to 92%, and the conversion of allyl phenyl ether was the highest (92%). Based on all the above, an obvious positive influence of both the presence of a fullerene moiety in the monomer structure and the mechanopolymerization on the increased porosity of the **FulCP** and the effectiveness of **FulCP-Pd** was demonstrated.

It worth mentioning that fullerene polymers have a wide range of applications for organic electronics, photovoltaics, and (photo)catalysis [160], including catalytic applications [161], energy storage and transfer [162], and photovoltaics [163]. 

**Figure 22:**
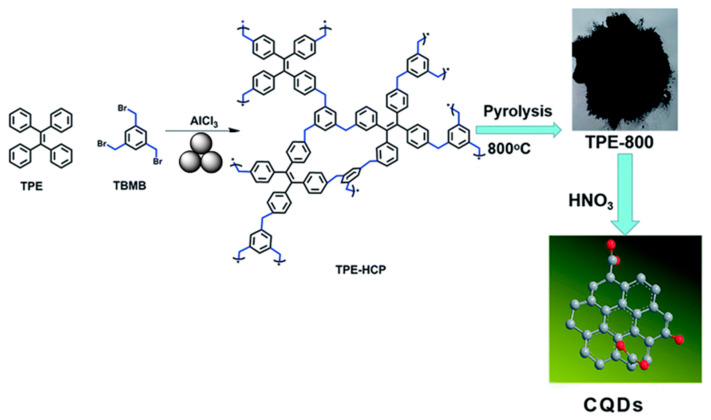
Mechanosynthesis of **TPEPP**. Reproduced with the permission of reference [164].

Pan and coworkers reported a dopamine-sensing system based on a mechanochemically synthesized tetraphenylethylene-based porous polymer (**TPEPP**) [164] (Figure 22). In the first step, the authors obtained a tetraphenylethylene (TPE)-based porous organic polymer by means of the reaction of catalytic amounts of FeCl_3_, 1,3,5-triformylbenzene, and 1,1,2,2-tetraphenylethene in a zirconium oxide milling vessel with zirconium oxide milling balls in a planetary mill at 500 rpm for 35 min to produce the target **TPEPP**. After that, the obtained **TPEPP** was carbonized, and a carbon quantum dot (**CQD**) was prepared. In the last step, a **CQD**/chitosan–graphene composite film electrode was constructed for the electrochemiluminescence-based determination of dopamine. The thus constructed electrode presented good repeatability and a high sensitivity to dopamine with a wide linear range from 0.06 to 1.6 μM. In addition, a satisfactory detection limit of 0.028 μM (S/N = 3) was achieved. Finally, the authors demonstrated the possibility of detecting the dopamine concentration in human fluids (namely in serum samples). 

### 2.8. Mechanochemical Post-Modification of Polymers

Mechanochemical methods are readily used for polymer post-modification and for the preparation of co-polymers. Below, some of the most representative examples are highlighted. 

**Figure 23:**
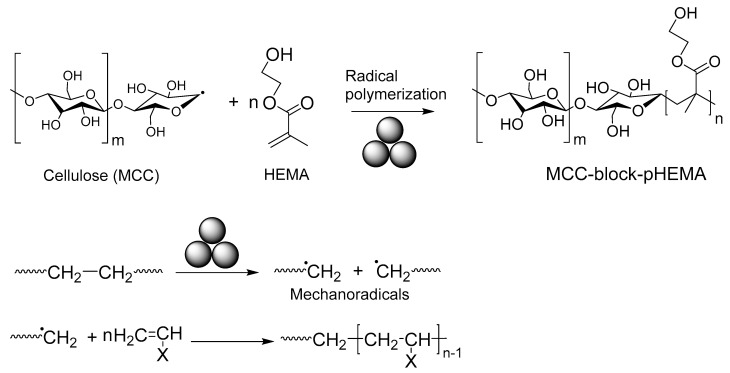
Mechanosynthesis of MCC-block-pHEMA.

Ohura and co-authors reported [165] (Figure 23) the synthesis of diblock copolymers of microcrystalline cellulose (**MCC**) and poly 2-hydroxyethyl methacrylate (**pHEMA**) produced by mechanochemical polymerization under vacuum and at room temperature. The tacticities of the HEMA sequences in the **MCC-block-pHEMA** varied according to the reaction time, namely the fraction of pHEMA in the MCC-block-pHEMA increased up to 21 mol% with increasing the fracture time (~6 h). According to the authors, cellulose acted as a radical polymerization initiator that was capable of controlling the stereoregularity. During the mechanosynthesis, the mechanical fracturing of the polymer produced free-radical chain-ends, and their recombination resulted in block copolymers.

**Figure 24:**
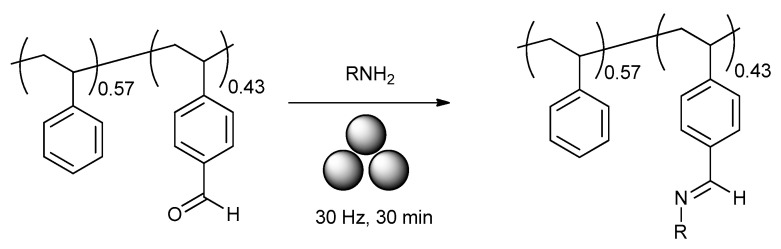
Mechanochemical post-modification of poly(stryrene-co-4-vinylbenzaldehyde).

Ohn and Kim reported [166] (Figure 24) the mechanochemical post-modification of poly(stryrene-co-4-vinylbenzaldehyde) via solid-state Schiff’s base formations with a series of amines and amine derivatives. In a typical case, polymer, amine, and ammonium carbamate salt were reacted in a stainless-steel jar with three stainless-steel balls (7 mm) at 30 Hz for 30 min. Regardless of the nature of the amine, a 98–99% conversion with a PDI of 1.16–1.33 was observed. 

**Figure 25:**
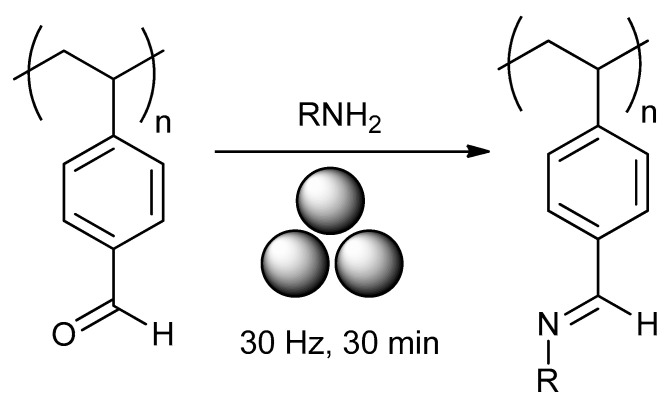
Mechanochemical post-modification of poly(4-vinylbenzaldehyde).

In addition to poly(stryrene-co-4-vinylbenzaldehyde), the mechanochemical post-modification of poly(4-vinylbenzaldehyde) (Figure 25) was carried out. 

**Figure 26:**
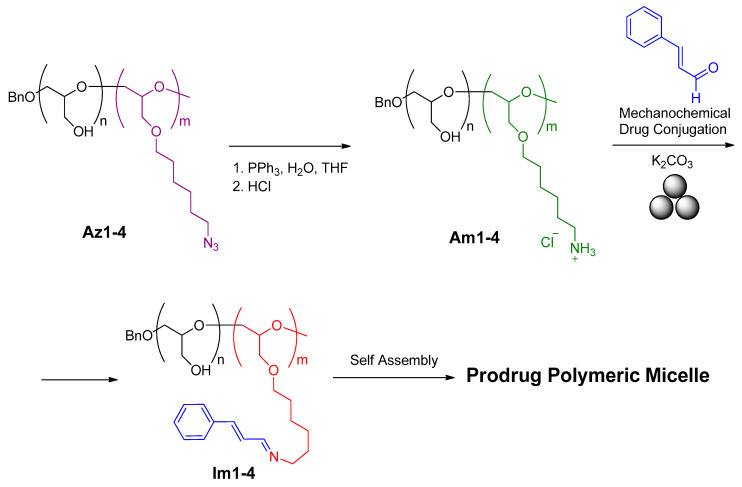
Mechanochemical post-modification of **AM1-4**.

In a similar way, Kim and coauthors [167] developed a mechanochemical approach for the post-modification of diblock copolyethers PEEGE-b-PAHGE obtained from monomers of ethoxyethyl glycidyl ether (EEGE) and azidohexyl glycidyl ether (AHGE) (Figure 26). The mechanochemical modification of the polymer-appended amino-functionality with a highly hydrophobic and potent anticancer agent, cinnamaldehyde, through an imine linkage. The resulting polymer–drug conjugates were further self-assembled into polymeric micelles, which was confirmed by dynamic light scattering and atomic force microscopy. In the obtained Schiff’s base-appended polymers, **IM1-4**, the *pH*-responsive cleavage of the imine linkages under acidic conditions led to the release of cinnamaldehyde with a concomitant disassembly of the polymeric micelles. 

**Figure 27:**
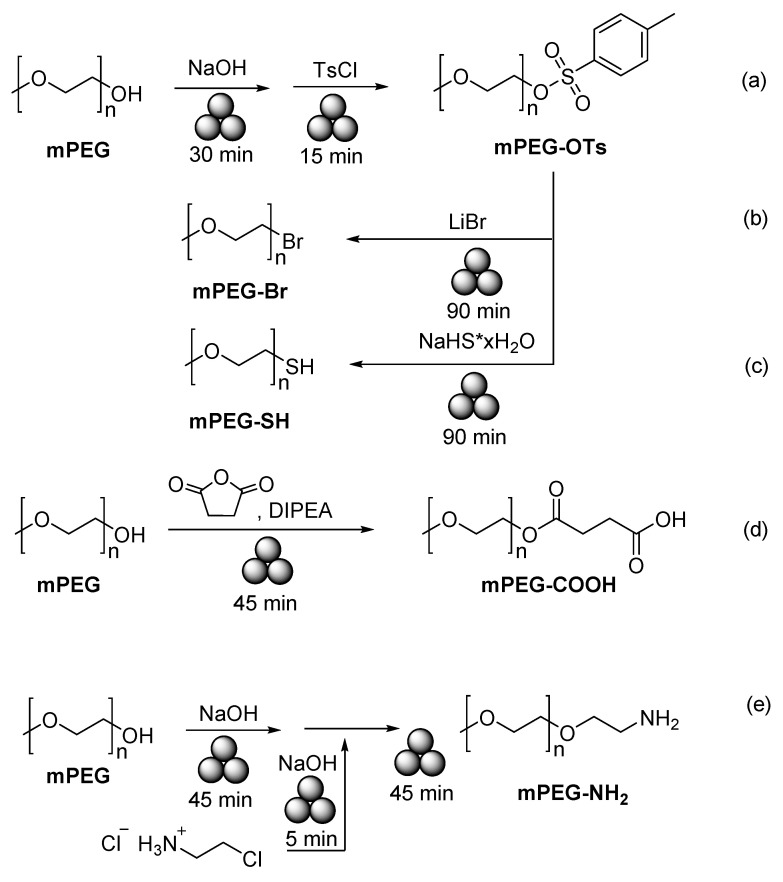
Mechanochemical ω-functionalization of **PEG**, tosyl (**a**), bromide (**b**), thiol (**c**), carboxylic acid (**d**), and amine (**e**).

Friščić and co-authors reported a solid-state mechanochemical ω-functionalization of poly(ethylene glycol) (PEG) with tosyl (Figure 27a), bromide (Figure 27b), thiol (Figure 27c), carboxylic acid (Figure 27d), and amine (Figure 27e) functionalities (Figure 27) in good-to-quantitative yields [168]. In the most typical case, a PEG polymer and the corresponding reagents were milled in a Teflon jar with one 10 mm Zr ball in a Retsch Mixer Mill 400 at 30 Hz for 15–90 min to produce the desired polymer. Depending on a nature of PEG, its molecular weights, and the type of functionality introduced, the reaction was completed in 15–90 min and provided the desired polymers in 42–99% yields (according to ^1^H NMR). 

**Figure 28:**
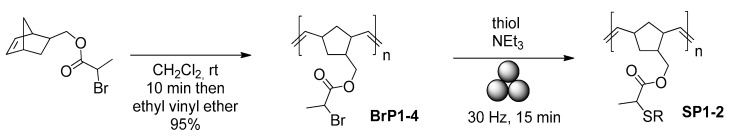
Mechanochemical post-modification with thiols.

Ashlin and Hobbs reported [169] a mechanosynthesis-assisted post modification of polymers **BrP1-4** with thiol moieties (Figure 28). In a typical case, the polymer and the corresponding thiol were milled in a stainless-steel grinding jar equipped with three stainless-steel grinding balls (7 mm) using a Retsch MM-400 ball mill at 30 Hz for 15 min to produce the thiol-containing polymers in up to a 95% yield. To prove the concept, the authors prepared a chloromethyl-functionalized-polymer by the co-polymerisation of styrene and 4-vinylbenzyl chloride, and, under similar conditions, the substitution of the chlorine atom of the benzyl chloride moiety with various thiols was also achieved in high yields. 

It worth mentioning that similar types of parent polymers could be obtained using ruthenium-alkylidene catalysts for ring-closing and cross-metathesis reactions under ball-milling conditions [170,171]. 

**Figure 29:**
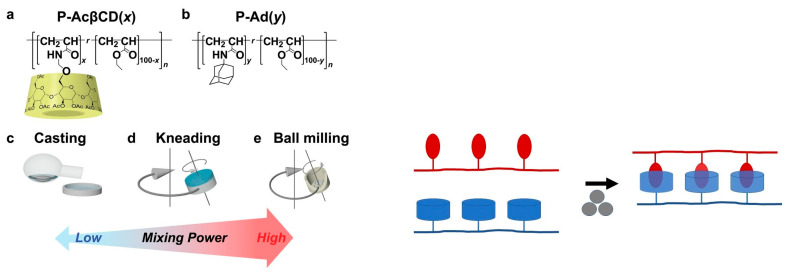
Mechanochemical post-modification via host–guest interactions, cyclodextrin (**P-AcβCD(x)**) moieties (**a**), adamantane (**P-Ad(y)**) moieties (**b**), ball milling (**e**), conventional method (**c**) and kneading (**d**). Reproduced with the permission of reference [172].

A quite rare example of the post-modification/co-polymerization of two polymers via host–guest interaction under mechanochemical conditions was reported by Park and co-authors [172] (Figure 29). In the first step, the authors prepared two polymers bearing cyclodextrin (**P-AcβCD(x)**) (Figure 29a) and adamantane (**P-Ad(y)**) (Figure 29b) moieties. In the second step, by mixing the host and guest polymers by planetary ball milling, the supramolecular host–guest-based polymer was obtained. According to the authors, the toughness of the supramolecular materials prepared by ball milling (way e) was approximately two-to-five times higher than that of supramolecular materials prepared by casting as a conventional method (way Figure 29c) or kneading (way Figure 29d), and during repeated ball-milling treatments, the obtained supramolecular polymers were able to maintain their mechanical properties. These materials are readily applicable as self-healable bulk materials and coatings, as their fractured pieces can be re-adhered within 10 min. 

## 3. Conclusions and Future Perspectives

In summary, mechanochemical synthesis has become a convenient tool for the construction of functional homo- or co-polymers of various types, as well as for the fast and efficient introduction of extra functionalities into terminal ends as well as side arms with a high degree of conversion of previously prepared polymers without rupturing the main polymer backbone. The main advantages of mechanosynthesis over the conventional solvent-based synthesis of functional polymers include the much shorter reaction times (from several minutes to several hours), the absence of the influence of the solvent (and its associated solubility problems for both the target polymer and starting monomers), the lower mass intensity, the higher mass productivity, and, finally, the much lower overall E-factors (due to absence of solvents). The most-accepted mechanism of mechanopolymerization involves the formation of short-lived mechanoradicals generated by mechanical force, which was confirmed in some publications by using radical traps, such as TEMPO [173]. Due to strong influence of the mechanical force in the reaction, the hardness and size/weight of the milling balls, as well as intensity of the ball-milling, are critical for achieving a high monomer conversion, and the best results were reported for tungsten carbide (WC) and zirconium oxide (ZrO) and for agate milling balls (pests) and with a milling speed/frequency higher than 300 rpm/5 Hz. The avoidance of solvents is beneficial for the mechanosynthesis of various organic porous polymers, which are hardly available by means of conventional solvent-based methods with a high porosity/surface area for gas separation or catalysis. Finally, mechanochemical synthesis is a convenient tool for the preparation of functional polymers by using industrial by-products, for instance, sulfur [174,175], industrial/post-consumer wastes [6,176], or agricultural wastes [177].

For polymer characterization, a set of common methods is usually used. The most important factor is the molecular weight of the functional polymer. Most often, gel permeation chromatography (GPC) [19,41,73] and size-exclusion chromatography (SEC) [40,42] are used for the estimation of molecular weights. The main limitation of DPC/SEC is the solubility of the analyzed polymer in organic solvents (most commonly DMF and THF). More rarely, MALDI-TOF analysis [51] or, if it is possible to end-cap the obtained polymer with ^1^H NMR- or IR-distinguishable end-groups, NMR- [54] or IR-based [178] end-group analysis are used. Additionally, ^1^H NMR analysis can be used for the estimation of monomer(s) conversion [40]. During mechanosynthesis, the heating of the milling balls and, as a result, the local overheating of the reaction media/obtained polymer is questioned in the literature [179]. Therefore, the thermal degradation of polymers, i.e., the maximum temperature at which a polymer can be manufactured and processed, is another important parameter and can be used for polymer analysis. To estimate the thermal stability of polymers, thermogravimetric analysis (TGA) for measuring polymer weight changes as a function of temperature and time, differential thermal analysis (DTA) for measuring the glass and other polymer transitions, or differential scanning calorimetry (DSC) for investigating the response of polymers to heating, such as the melting of a crystalline polymer or the glass transition, can be used. For porous functional polymers such as those used for catalytic applications and gas separation/storage, the contact surface area and pore (voids) size is important. To estimates these values, the Brunauer–Emmett–Teller (BET) surface area can be calculated for the theoretical estimation of the physical adsorption of gas molecules (most commonly N_2_) on a solid surface of the polymer [135,140,143,157]. 

Regarding future perspectives, one can mention the following. Among the TM-catalyzed mechanochemical approaches to producing functional polymers, Pd-catalyzed processes, which have been widely explored for small molecules [180], have so far only been reported by a few cases of Suzuki cross-coupling reactions [52,84,85,98]. So, in the near future, one might expect the interest in mechanopolymerization reactions based on the Buchwald, Stille, or Sonogashira cross-coupling protocols to grow. 

Finally, in terms of closed-loop economic systems, to solve the end-of-use problem for synthetic polymers, one needs to either design polymers composed of a certain type of dynamic bonds that are capable of effective bonding and reversible cleaving and/or to develop of efficient ways for the polymers to depolymerize into monomers. For the first approach, to break the dynamic bonds, high temperatures are usually required and, in some cases, the thermo-degradation of polymers may occur, which may influence their mechanical properties [181,182]. We mentioned [71] one example above of the mechanosynthesis of diketoenamine-bond-connected polymers for ready mechanopolymerization/depolymerization at room temperature. Very recently, another type of room-temperature-recyclable polymer containing dynamic maleic acid tertiary amide bonds was reported [183]. 

For commercial polymers, their thermal degradation, except their monomers, produces multiple decomposition products, including oligomers and char [184,185,186,187,188]. In addition, ball-milling-assisted depolymerization might be a greener alternative to the above-mentioned approaches, which was recently suggested in reports on the mechanochemistry-assisted depolymerization of polyethylene terephthalate (PET) [189] and polystyrene [190]. 

## Data Availability

The data presented in this study are available on request from the corresponding author.
